# Heat Transport Control and Thermal Characterization of Low-Dimensional Materials: A Review

**DOI:** 10.3390/nano11010175

**Published:** 2021-01-13

**Authors:** Alexandros El Sachat, Francesc Alzina, Clivia M. Sotomayor Torres, Emigdio Chavez-Angel

**Affiliations:** 1 Catalan Institute of Nanoscience and Nanotechnology (ICN2), CSIC and BIST, Campus UAB, Bellaterra, 08193 Barcelona, Spain; francesc.alzina@icn2.cat (F.A.); clivia.sotomayor@icn2.cat (C.M.S.T.); emigdio.chavez@icn2.cat (E.C.-A.); 2 ICREA, Passeig Lluis Companys 23, 08010 Barcelona, Spain

**Keywords:** phonon engineering, nanoscale thermal transport, thermal characterization, semiconductors, 2D materials

## Abstract

Heat dissipation and thermal management are central challenges in various areas of science and technology and are critical issues for the majority of nanoelectronic devices. In this review, we focus on experimental advances in thermal characterization and phonon engineering that have drastically increased the understanding of heat transport and demonstrated efficient ways to control heat propagation in nanomaterials. We summarize the latest device-relevant methodologies of phonon engineering in semiconductor nanostructures and 2D materials, including graphene and transition metal dichalcogenides. Then, we review recent advances in thermal characterization techniques, and discuss their main challenges and limitations.

## 1. Introduction

Advances in the electronics industry have led to an increased need for novel approaches to thermal management to improve devices performance and reliability, by controlling the dissipation of the energy generated in the devices. In particular, the possibility of controlling heat propagation by engineering the phononic properties of the fundamental components is of great interest in nanoelectronics—where heat dissipation will play a major role in determining the performance of high-density nanoscale circuits —or in thermoelectric materials—where materials with low thermal conductivities are desired. The main heat carriers in these materials are phonons, thus understanding and controlling phonon transport are issues highly connected with the successful development of low-power electronics and efficient thermoelectric energy harvesting. 

However, with the continuous miniaturization of electronic devices reaching physical limits, heat transport and thermal management are becoming increasingly more challenging. For instance, the characteristic dimensions of electronic components have become comparable to the phonon mean free path (MFP), which inevitably increases the power density and complicates heat removal [1,2]. In addition, the large density of interfaces, contacts, and boundaries that appear at extremely small length scales in today’s electronics indicates the importance of further optimizing nanoscale thermal characterization tools. Advances in measurement techniques together with theoretical efforts have enabled a better understanding of novel heat transport mechanisms, e.g., hydrodynamic phonon transport, coherent and ballistic transport, thermal localization, and finally phonon propagation at the nanoscale, opening exciting prospects for thermal investigations of materials even at the atomic level [3,4]. In parallel, progress in material growth and nanofabrication have enabled remarkable advances in thermal transport engineering. The concept of phonon engineering has been employed in various nanomaterials during recent decades, showing its potential in thermal management. 

Here, we review recent works that have demonstrated efficient ways to control heat conduction in nanomaterials by phonon engineering, focusing mainly on semiconductor nanostructures and two-dimensional materials. Then, we review recent advances in the most commonly used experimental techniques that have enabled heat transport measurements and thermal characterization at the nanoscale. We also discuss the main limitations and challenges of these techniques and suggest future directions for nanoscale thermal characterization.

## 2. Engineering the Phonon Thermal Conduction in Semiconductor Nanostructures and 2D Materials

In semiconductor and insulators, the dominant carriers of heat conduction are lattices waves or phonons. A phonon is a quasi-particle which represents quantized modes of the vibrational energy of an atom or group of atoms in a lattice. Considering that phonons are pseudo-particles, it is possible to associate energy ħω (where ħ is the reduced Planck’s constant ħ = h/(2π) and ω is the angular frequency) and a pseudo-momentum p = ħq (where q is the wavevector), which obey Bose–Einstein statistics [5,6]. The wavelength dependence of the phonon energy can be represented as a dispersion relation, i.e., a relationship between the phonon frequency and its wavevector. The slope of a dispersion relation curve determines the phonon group velocity. 

The ability to transport heat is denominated thermal conductivity. It plays a fundamental role in the design and performance of the technological devices. The calculation of the thermal conductivity (*k*) in semiconductor material requires the knowledge of three major frequency-dependent parameters, namely, specific heat (*C_V_*), phonon group velocity (v_g_), and phonon mean free path (Λ). Finally, the expression for thermal conductivity from the kinetic theory of gases is given by: *k* = *C_V_*·v_g_·Λ. 

A major limitation to determine *k* is the knowledge of mean free path Λ = v_g_/τ, where τ is the effective or total phonon lifetime. In general, τ is estimated using the Matthiessen’s rule assuming that each scattering mechanism is independent of each other. The phonon lifetime is mainly limited by: phonon-phonon scattering (τ_pp_), impurity scattering (τ_I_) and boundary scattering (τ_B_). The latter is pronounced in low-dimensional materials due to the dimensionality confinement, which results in modified heat transport properties. The possibility of tuning the thermal conductivity of low-dimensional materials via phonon engineering is of high importance and might lead in multiple breakthroughs (e.g., high figure of merit, improved energy efficiency).

### 2.1. Semiconductor Nanostructures

Modifications of the dispersion relation have a direct impact on the acoustic phonon properties of nanostructures, such as phonon group velocity [7], polarization and density of states. These can usually be induced either through boundary conditions in the individual nanostructures, e.g., free-standing nanowires (NWs) or thin films, or via periodic boundary conditions, e.g., superlattices (SLs) and phononic crystals (PnCs). In principle, heat transport in such nanostructures decreases either due to classical size effects or phonon confinement effects. The first is related to increased phonon-boundary scattering and is pronounced when the characteristic dimensions of the nanostructures are comparable to the phonon MFP [8]. When nanostructures dimensions are in the order of or smaller than the phonon wavelength, phonon confinement or coherent effects appear, modifying dispersion branches, which in turn modifies the group velocity, phonon density of states, and phonon lifetime [9,10,11]. At room temperature, the impact of phonon confinement on the thermal transport is almost negligible. Instead, the decrease of the thermal conductivity is mainly attributed to diffuse scattering of phonons at the boundaries. Although this mechanism has been widely explored and exploited, several works propose the use of the phonon confinement effect as a mean to control the heat flow [12,13].

The real impact of the phonon confinement on thermal transport at room temperature has only been observed using superlattices [14,15]. However, in most cases nanofabrication processes result in nanostructures with length scales larger than the phonon wavelength of the dominant heat carriers (at room temperature <5 nm) and limits the observation of confinement effects. Cryogenic temperatures (T < 10 K) can overcome this problem [16,17]. In the next sections we present recent experimental works that have demonstrated efficient heat transport control in semiconductor nanostructures.

#### 2.1.1. Membrane-Based Structures

In membrane-based structures, the reduction of in-plane thermal conductivity (*k*) due to phonon-boundary scattering has been clearly demonstrated in thermal transport experiments in silicon layers of different thickness, performed over a large range of temperatures [18,19,20,21]. The results from these studies showed that the thermal conductivity of Si can be effectively tuned by decreasing its thickness. In parallel, experimental works have demonstrated that the fabrication of Si thin films with two-dimensional periodic patterning, i.e., phononic crystals (PnCs), is an efficient way to modify the phonon spectrum, control heat conduction and improve the thermoelectric efficiency [22,23]. 

**Figure 1:**
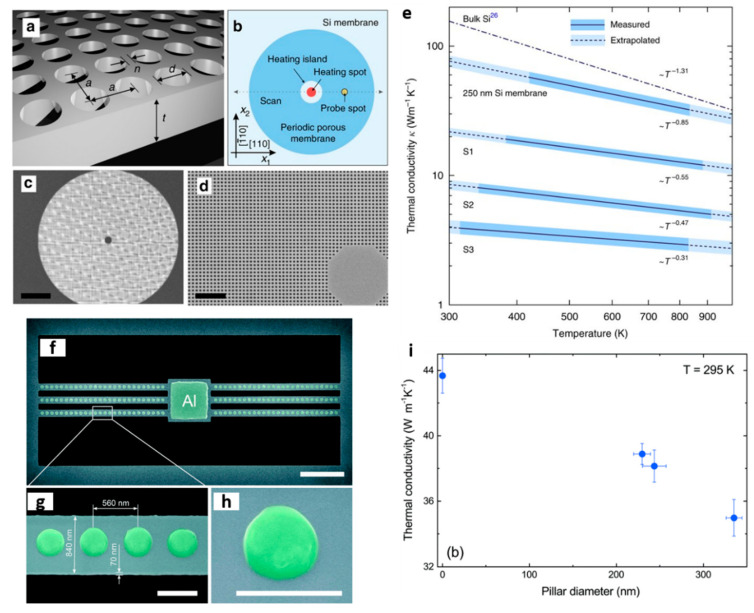
Phonon engineering in membrane-based structures. (**a**) Schematic of a hole-based PnC—square lattice of cylindrical holes in a 250 nm thick suspended membrane, where d is the hole diameter, a is the lattice parameter, and n is the neck size. (**b**) Schematic of a sample design showing relative laser heating and probing positions and (**c**,**d**) scanning electron microscope images of a PnC with a = 250 nm and d = 140 nm. Scale bars in (**c**,**d**) are 20 and 2 μm, respectively. (**e**) Thermal conductivity of hole-based PnCs as a function of temperature and filling fraction *S* with S1 = 0.159, S2 = 0.246 and S3 = 0.332. (**f**,**g**) SEM images of a pillar-based PnC—Si nanobeam with one-dimensional arrays of pillars with a period of 560 nm and pillar base diameters of 229.5, 243.5 and 335 nm and (**h**) SEM image of a single nanopillar. Scale bars are (**f**) 5 µm and (**g**–**h**) 500 nm. (**i**) Thermal conductivity of different nanobeams as a function of pillar diameter at 295 K. (**a**–**e**) reproduced with permission from [24]. Copyright Springer Nature, 2017. (**f**–**i**) Reproduced with permission from [28], Copyright Royal Society of Chemistry, 2017.

Recent thermal transport studies have shown that the in-plane thermal conductivity of silicon and its temperature dependence can be effectively reduced and tuned by patterning periodic arrays of holes [24,25,26] or arrays of pillars [27,28,29]. In silicon membranes with patterned arrays of holes (see Figure 1a–d) a strong reduction of ~90% of the thermal conductivity was found compared to unpatterned Si membranes of equal thickness. Figure 1e displays the thermal conductivity of PnCs with different filling fraction. At room temperature the reduction of the thermal conductivity was attributed mainly to the shortening of the phonon mean free path due to diffuse (incoherent) phonon-boundary scattering. Although the increase of the surface-to-volume ratio leads to increased boundary scattering, at higher temperatures the phonon–phonon scattering dominates over the boundary scattering. This is observed through the smaller relative reduction in *k*, compared to room temperature. The impact of coherent phonon scattering was found to be significant in the thermal conductivity reduction of similar structures only at low temperatures, where thermal phonon wavelengths become longer and comparable with the period of the holes [30,31,32].

In pillar-based PnCs the reduction of the thermal conductivity was weaker in comparison with the hole-based PnCs while coherent effects were found to be insignificant even at low temperatures. This was observed for pillar-based PnCs fabricated by patterning Al nanopillars of different diameters on suspended Si nanobeams [28]. Figure 1f–h shows the geometry of the investigated Si nanobeam with one-dimensional arrays of pillars with a period of 560 nm and various pillar diameters. The thermal conductivity of these structures at room temperature was observed to decrease with increasing diameter, with a maximum thermal conductivity reduction of approximately 20% (see Figure 1e). The authors attributed this behavior to the increased phonon scattering at the pillar/beam interface due to the intermixing of aluminum and silicon atoms. The same group later fabricated nanopillars on suspended silicon membranes and investigated the impact of nanopillars on the thermal conductivity at low temperatures (4–300 K) [29]. They found the thermal conductivity reduction caused by the nanopillars to be approximately ~16%, which was attributed mainly to incoherent phonon boundary scattering. It is interesting to note that although the rate of the thermal conductivity reduction in these structures was much lower than the hole-based PnCs, the electrical conduction remained unaffected, or even increased, since no volume removal was required.

Other phononic structures have been fabricated by introducing short-range positional disorder in PnCs, which showed similar values of the thermal conductivity at room temperature compared with the fully periodic structures [16,33]. Although the phonon spectrum in the GHz range may be modified, these works evidenced that at room temperature, thermal transport is mainly diffusive (particle-like) and dominated by phonons in the THz range. 

Since phonons are intrinsically waves, the control over their coherence can open fundamentally new routes for manipulating the heat flow. Venkatasubramanian was one of the first who discussed about coherent effects on thermal measurements in superlattices (SLs). He presented a physical model to understand the reduction of the *k* based on the coherent backscattering of phonon waves at the superlattice interfaces [34]. Since then, the coherent concept was adopted by several authors to explain thermal conduction processes in superlattices and phononic crystals [35]. However, the interpretation of coherent thermal transport is still under debate and the experimental reports still remain inconclusive [36]. Some experimental reports that claimed coherent effects [37,38,39] have been contrasted by numerical simulations [40,41]. Concluding that some of these claims could be explained by particle-based models without considering coherent phonon transport [40,41].

Part of these controversies comes from the nature of the coherent transport in the context of thermal transport which is not well understood. In general, coherence involves a measurable phase-dependence between waves over a given time interval, e.g., the interaction of monochromatic waves. However, this notion cannot be applied directly in case of heat conduction, which involves all the thermally excited phonons in a structure [14]. Latour et al. tackled this problem by treating the phonon-coherence length in terms of correlation functions in superlattices [42]. The discussion about coherent effect in superlattices will be given in the Section 2.1.3. 

The sample quality is also another important parameter to take into account to observe coherent effects. The structures have to have periodicities in the order of the wavelength of the dominant thermal phonons (few nm) with atomically smooth surfaces (or interfaces) to avoid diffusive scattering of the heat carriers. For the case of silicon, the dominant phonon wavelength at room temperature is 1–2 nm [41]. On the other hand, the present state of the art in nanofabrication can produce patterned structures with dimensions down to several tens of nanometers with block-copolymer technologies and hundreds of nanometers via a top-down approach [43]. Such dimensions can tailor the dispersion relations of phonons in the GHz range with a poor contribution to the thermal properties at room temperature [33]. Lee et al. demonstrate that phonon coherence is negligible in the thermal transport of silicon nanomeshes with periodicities ≥100 nm and T > 14 K. Xiao et al. also found a negligible contribution of wave effects in the total thermal resistance of Si thin film with increased rows of nanopores with temperatures ranging from 85–300 K [44]. 

On the other hand, at lower temperatures, Zen et al., demonstrated the impact of the coherent effect in the thermal transport in patterned silicon nitride membranes in the sub-Kelvin regime. They showed the direct correlation between the thermal conductance, calculated from the modified phonon dispersion relation, and experimental measurements [32]. Maire et al. measured the reduction in the *k* in a patterned Si phononic crystal at 4 K. They claimed that the presence of phonon interference is the origin of the reduction in *k* of a phononic crystal with an ordered array of holes as compared to the thermal conductivity of structures with randomly positioned holes [16].

#### 2.1.2. Nanowires

Tuning phonon properties and heat conduction via phonon engineering has been demonstrated in NWs consisting of different materials, shapes, geometries and composition. The influence of diameter of NWs on the phonon thermal conductivity at room temperature has been thoroughly investigated in previous studies [45,46,47,48]. In these experiments, classical size effects were dominant and the thermal conductivity of the NWs was found to be suppressed by almost two orders of magnitude compared to their bulk counterparts, mainly due to the increased phonon boundary scattering. The dependence of the thermal conductivity on diameter is still valid at high temperatures as has been recently demonstrated by Lee et al. [48]. Additionally, in this work the authors showed an increasing contribution of high-frequency phonons as the temperature increases and the NW diameter decreases.

Furthermore, recent works have experimentally demonstrated ballistic heat conduction in Si, SiGe, and GaN NWs of different lengths at room temperature [49,50,51]. The length-dependent thermal conductivity measured in these studies showed that ballistic heat conduction can be preserved at room temperature for several micrometer length wires. For instance, Vakulov et al. [51] showed that in 25 nm diameter GaN NWs a room-temperature ballistic heat flow persist at least 15 μm. Such evidence showed the great potential of semiconductor NWs to be used for improved thermal management in applications such as phonon transistors [52,53] and computer chips, where rapid heat removal is required.

**Figure 2:**
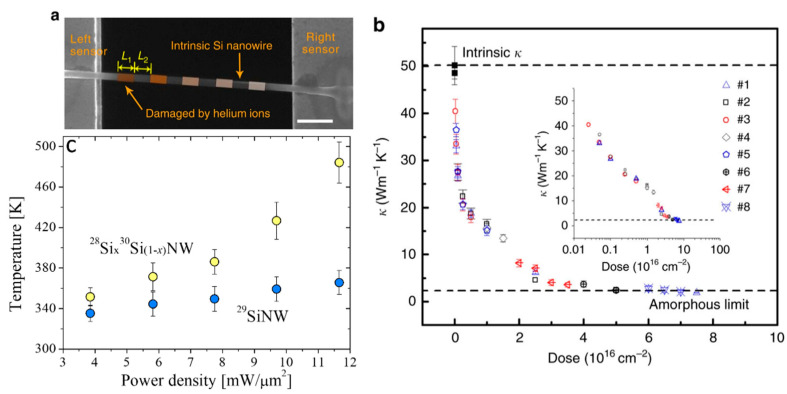
Phonon engineering in nanowires. (**a**) SEM image of a Si NW damaged by helium ions (sample #1). The portions colored orange denote the parts damaged by helium ions; the uncolored portions denote the intrinsic Si NW. Scale bar, 1 mm. (**b**) Measured *k* of samples #1–#8 versus dose. Inset: the same data plotted on a logarithmic scale. The solid black square denotes the *k* of intrinsic NWs (namely, with zero dose). (**c**) Plot of the measured power density as a function of the laser heating for different isotopically engineered Si NWs. (**a**,**b**) Reproduced with permission from [56]. Copyright Springer Nature, 2017. (**c**) Adapted from Mukjerjee et al. [57].

In parallel, different methods to further modulate the thermal conductivity of NWs have been proposed such as the fabrication of core-shell NWs. For example, a strong thermal conductivity reduction was found in Si and SiGe alloy NWs with diameters of few tens of nanometers, indicating the important effect of the core–shell interface on phonon transport [54]. Juntunen et al. also found up to ~60% reduction of the thermal conductivity of GaAs NWs coated with AlAs shells [55]. A different study showed that the *k* along a single Si nanowire can be tuned (between crystalline and amorphous limits) through selective helium ion irradiation with a well-controlled dose [56]. Figure 2a displays a SEM image of a single Si nanowire, which was irradiated at different positions with well-controlled helium ion doses. Figure 2b shows the reduction in *k* as a function of the helium ion doses, where a clear transition from crystalline Si to amorphous phase can be observed at a dose between 1.5 × 10^16^ and 2.5 × 10^16^ cm^−2^. 

More recent experimental studies demonstrated that manipulation of crystal phase, isotope composition and mass disorder are effective ways to control heat transport in silicon NWs. For instance, Mukherjee et al. showed that isotopically mixed metal-catalyzed ^28^Si_x_^30^Si_1−x_ NWs exhibit enhanced phonon scattering and approximately 30% decreased thermal conductivity induced by mass disorder in comparison with isotopically pure ^29^Si NWs [57]. Figure 2c shows the measured power density as a function of the laser heating for the two types of NWs, which was used together with a model to extract the local temperature and thermal conductivity of the NWs. The same authors later found that the thermal conductivity of Si NWs with tailor-made isotopic compositions can be reduced by up to ~40% relative to that of isotopically pure NWs [58]. The lowest *k* value was found for a rhombohedral phase in isotopically mixed ^28^Si_x_^30^Si_1−x_ nanowires with composition close to the highest mass disorder. Similarly, the authors used the same methodology to extract the thermal conductivity of the NWs.

#### 2.1.3. Superlattices

**Figure 3:**
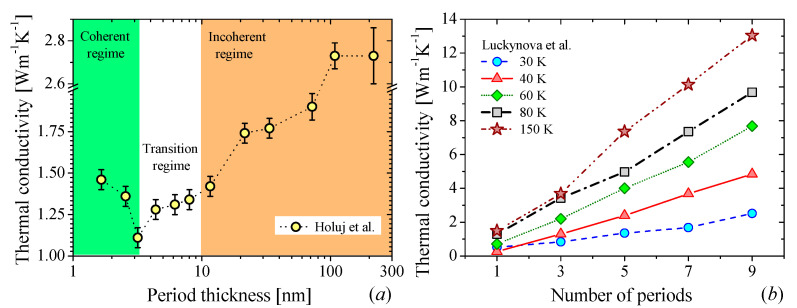
Phonon engineering in superlattices. Experimental *k* as a function of: (**a**) period thickness of (TiNiSn):(HfNiSn) half-Heusler superlattices, (**b**) number of periods of GaAs/AlAs superlattices. Adapted from Holuj et al. [60] and Luckynova et al., [14], respectively. (**a**) The crossover between coherent-incoherent (wave-particle) regimes is observed as a minimum in *k* vs. *d_SL_*, while in (**b**), the linearity of the *k* vs. *N* suggests that phonon heat conduction is coherent.

The first attempts to manipulate the wave nature of phonons were carried out by using alternating thin layers of dissimilar materials to realize a super periodicity of atomic position, i.e., a superlattice (SL). Due to the possibility to modify the dispersion relation as well as to create miniband and minigaps, stop bands and acoustic mirrors, the thermal transport community envisioned a very large potential to control the heat propagation with SLs [59]. The thermal transport in nanoscale SLs shows a crossover between coherent and incoherent phonon transport along the layered axis. The transition depends on the period thickness (*d_SL_* = *d*_1_ + *d*_2_, where *d*_1_ and *d*_2_ are the thickness of each layer) and the coherent length of the phonons. The crossover occurs when the interface density, 1/*d_SL_*, is large enough to limit the propagation of high frequency phonons (particle-like) so that the thermal transport is governed by low frequency phonons (wave-like). The transition between coherent-incoherent (wave-particle) transport is observed as a minimum in the *k* as a function of *d_SL_* [15,34] as is shown in Figure 3a. Although this behavior was predicted in 2000 [34], this observation has been hidden probably by the low quality of the interfaces, which destroys the otherwise perfect periodic system, disallowing coherent phonon transport. Recently, Ravichandran et al. [15] presented the first unambiguous experimental demonstration of this crossover using epitaxial perovskite-based SLs. Luckyanova et al. [14] presented another fingerprint of coherent thermal transport, namely, a linear dependence of *k* with respect to the number periods *N* (see Figure 3b). This behavior arises when, in the coherent regime, the phonon mean free paths are equal to the total SL thickness, resulting in a linear dependence between *k* and *N*.

**Figure 4:**
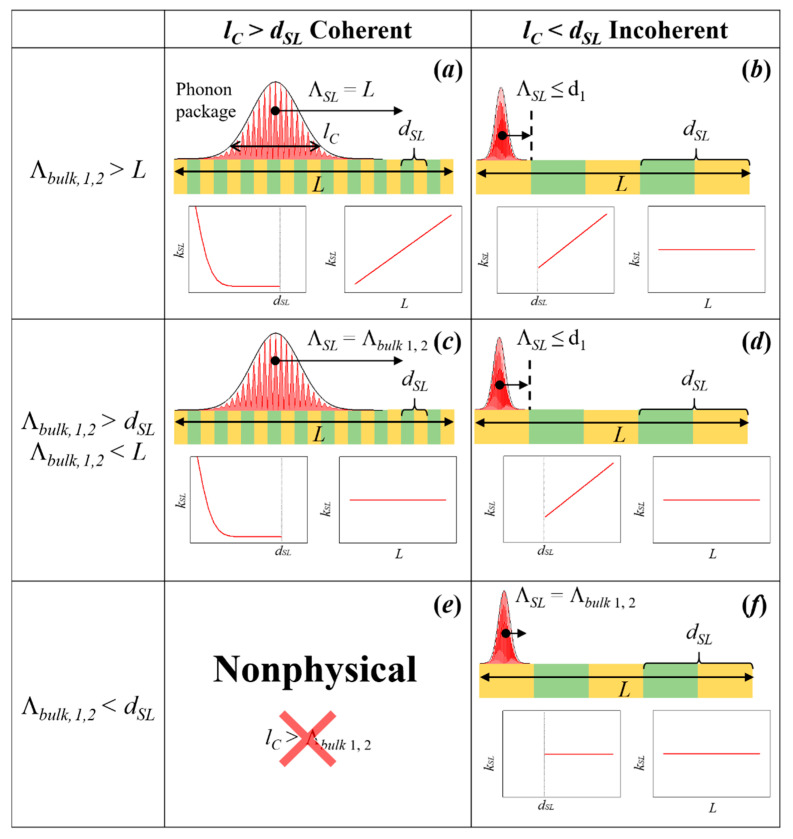
(**a**–**f**) Schematic representation of coherent and incoherent thermal transport in superlattices (adapted from Latour et al. [42]).

As we mentioned above, the concept of coherency cannot be applied directly in case of heat conduction because the thermal transport involves all excited phonons of the structure. However, Latour et al. [42] showed that coherence can be formalized in other physical fields as correlation, e.g., the spatial coherence of the light can be expressed in terms of spatial correlations of electromagnetic fields. Inspired by this theory, Latour et al. extended this concept to the thermal phonons in superlattices. They postulated that the spatial phonon coherence length (*l_C_*) can be related to the spatial correlations of the atomic displacement fluctuations at equilibrium. The authors noted that if two atoms separated by a distance *l* and oscillating with a given phase and frequency (i.e., nonrandom), their motion is correlated. Hence, the finite spatial extension in which this correlation remains preserved is defined as spatial coherence length *l_C_*. This correlation arises from the presence of phonon wave packets composed by atoms vibrating in phase. Using this approach, the authors were able to distinguish different regimes of heat conduction characterized by the coherent length (*l_C_*), mean free path of the packet (Λ), period thickness (*d_SL_*) and total thickness of the superlattice (*L*). Then, the nature of the thermal transport will be given by the combination of these parameters as is shown in Figure 4. From the figure we can note that when *l_C_* > *d_SL_* (Figure 4a,c), the phonon transport is coherent. However, *l_C_* cannot be larger than the bulk mean free path (*l_C_* ≥ Λ*_bulk_*, see Figure 4e). The wave package cannot travel a distance larger or equal to its spatial extension without scattering, i.e., it is a nonphysical phenomenon. For each of the rest of the cases shown in the figure, two trends for the thermal conductivity are depicted: one as a function of the *d_SL_* with a constant *L* and as a function of *L* with constant *d_SL_*. The crossover of thermal conductivity happens in Figure 4b,d,f. In these cases, the thermal conductivity becomes independent of the system size and increases with the SL period.

To observe coherent thermal transport, it is necessary that the incoming thermal wave retains its phase after it has been reflected or transmitted across the interface. This implies that the scattering mechanisms should not be purely diffusive, otherwise the phase information will be destroyed. Consequently, the presence of atomically smooth interfaces becomes mandatory. Although numerical simulations carried out by Qui et al. found the same linear dependence in rough periodic and aperiodic Si:Ge SLs [61], the results of their simulations were associated to the low interface densities and weak disorder scattering. Under these conditions, the dominant thermal phonons would not be affected by the disorder and could ballistically transverse the SLs regardless of aperiodicity or interface roughness. Similar results were found by Wang et al. [62] and Chakraborty et al. [63] in rough periodic SLs and random multilayer structures (RML) made of artificial atoms. Both simulations showed the same linear-like behavior of *k*_⊥_ vs. *N*. However, the absence of a minimum in *k*_⊥_ as a function of *d_SL_* in the simulations performed by Wang et al. suggest a ballistic phonon transport rather than coherent effects [62].

On the other hand, the introduction of very small-periods (10s of nm) have also shown a large impact on lowering *k*. Values close or smaller than the amorphous limit of one (or both) component of the SLs have been reported. Costescu et al. [64], Pernot et al. [65], and Chavez-Angel et al. [66] measured cross-plane thermal conductivity values (*k*_⊥_) below the amorphous limit of Al_2_O_3_, Si, and HfNiSn in Al_2_O_3_:W, SiGe:Si and HfNiSn:TiNiSn SLs, respectively. Niemelä et al. [67] also overtook the amorphous limit of TiO_2_ using organic-inorganic (TiO_2_):(Ti–O–C_6_H_4_–O) SLs. 

Ultralow values of *k* were also reported by Juntunen et al. [68] in aperiodic Si:Ge SLs. The authors explained their observation in terms of wide range Anderson localization, which leads to a destructive interference of coherent phonons and consequently a drastic reduction of *k* by quenching the wave transport under structural disorder. Phonon localization was also reported by Luckynova et al. [69] using GaAs/AlAs superlattices with 8 and 25% of ErAs nanodots randomly distributed at the interfaces. They observed peaks in the normalized *k* of SLs as function of number of periods at 30 K and 50 K for 25% ErAs sample. Their observations were supported by theoretical calculations and explained in terms of a new heat conduction mechanism related to the presence of phonon localization in these SLs.

### 2.2. Two-Dimensional Materials

#### 2.2.1. Graphene

The emergence of graphene has provided with a platform for the study of 2D phonon transport [70,71,72,73] and, at the same time, it’s extremely high thermal conductivity has driven applications in thermal management [74] and energy conversion [75]. Experimental studies have shown the possibility of tuning graphene’s thermal properties with different methods such as the control of isotope composition [76], metal deposition [77], introduction of defects [78,79,80], and orienting the grain size in polycrystalline graphene [81,82,83].

The development of methods for labelling [84] and growing [85] large grain-size monolayer graphene with regions of different concentrations of ^12^C and ^13^C has made possible the study of the impact of isotope concentration on the thermal properties. It was found that the *k* of suspended isotopically pure ^12^C (0.01% ^13^C) graphene can reach values higher than 4000 W m^−1^ K^−1^ close to room temperature (*T*≈320 K), which is more than a factor of two higher than the value of *k* in graphene sheets with an equal composition of ^12^C and ^13^C [76]. In addition, Malekpour et al. [78] found that as the defect density in suspended graphene increased from 2.0 × 10^10^ cm^−2^ to 1.8 × 10^11^ cm^−2^ the thermal conductivity decreases more than a factor of ∼4 near room temperature. The defects in this work were induced by irradiating graphene with a low-energy electron beam (20 keV). A different study also used oxygen plasma treatment to induce defects in suspended graphene and reduce its thermal conductivity more than 90% [80].

**Figure 5:**
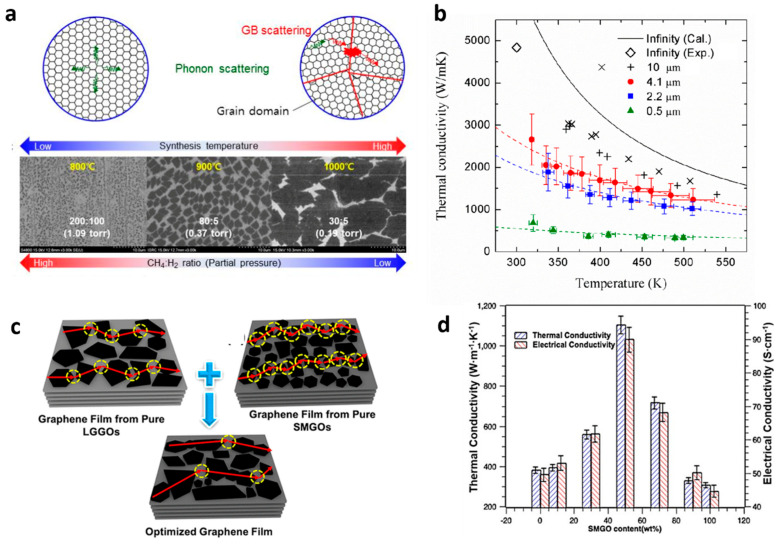
Phonon engineering in graphene. (**a**) Schematic illustration of the scattering mechanisms in polycrystalline graphene, i.e., phonon-phonon scattering and grain boundary scattering, and SEM images of samples with different nucleation densities. (**b**) The *k* as a function of the measured temperature for suspended graphene samples with grain sizes of 0.5, 2.2 and 4.1 nm. The symbol “◇” represents the *k* of exfoliated graphene. The *k* of “X” were measured for the suspended graphene on the hole of 9.7 μm in air and the *k* of “+” were measured for the suspended graphene on the hole of 8 μm in vacuum condition. (**c**) Schematics of the structure of the graphene films with different sized graphene oxides (large and small size graphene oxide: LGGO and SMGO, respectively) and (**d**) thermal and electrical conductivities of the graphene oxide films with different contents of small-sized graphene oxides (SMGO). (**a**,**b**) Reproduced with permission from [82]. Copyright American Chemical Society, 2017. (**c**,**d**) Reproduced with permission from [89]. Copyright American Chemical Society, 2020.

Moreover, the CVD method allows the growth of polycrystalline suspended single-layered graphene with controlled grain sizes by changing growth conditions (cf. Figure 5a) [82]. The *k* of the polycrystalline suspended graphene samples was found to decrease with decreasing grain size with a reduction up to a factor of ~5 at 300 K for grain sizes of 0.5 µm. In addition, there is an evident vanishing of the *k* vs. *T* dependence with decreasing grain size (cf. Figure 5b). Here, and similarly to the effect seen in Figure 1e for Si PnCs, the increased phonon boundary scattering with decreasing grain size competes with the temperature dependent phonon-phonon scattering as mechanism to reduce the thermal transport. Since the earliest measurements on graphene, it is well known that the boundary interaction between graphene and an adjacent dielectric such as SiO_2_ [86] has a large degradation effect on the thermal conductivity. The drastic reduction was attributed to the damping of the acoustic phonons of graphene in general, and of the flexural acoustic phonons in particular, owing to the scattering in the graphene-SiO_2_ rough interface and the symmetry breaking by the presence of the substrate [87]. The suppression of the in-plane thermal conductivity is even more drastic when graphene is encased within silicon dioxide layers, showing a thermal conductivity value below 160 W m^−1^ K^−1^ at room temperature [88].

Other works have reported the use of hydrogen-bonded graphene-polymer interfaces [90] or functionalized self-assembled monolayers on graphene [91] to enhance the thermal boundary conductance (TBC) up to an order of magnitude. In addition, graphene-polymer composites with enhanced cross-plane thermal conductivity have been successfully engineered, showing their potential to be used as thermal interface materials [92]. Moreover, Kim et al. measured significant changes in the TBC of graphene-metal interfaces by generating physical and chemical defects [93] while Hopkins et al. used chemical adsorption on the graphene surface through plasma oxygen in order to control the heat flow across metal-graphene interfaces [94]. The heat transport across Al/graphene interfaces increased by a factor of ~2 after the oxygen exposure of the graphene due to the enhancement of the bond strength between the Al and graphene atoms. 

Furthermore, thermal measurements on graphene laminate films on polyethylene terephthalate substrates have also indicated that the average size and the alignment of graphene flakes on the substrate are key parameters defining the heat conduction [95]. Finally, thermally conductive graphene films with an in-plane thermal conductivity up to 1102.62 W m^−1^ K^−1^ have recently been produced by simple chemical reduction of graphene oxide [89]. The structure of the graphene films with different sized graphene oxides is illustrated in Figure 5c. The graphene films with equal percentage of small (SMGO) and large sized graphene oxides (LSGO) showed minimized phonon scattering and maximum *k*, as is shown in Figure 5d.

#### 2.2.2. Transition Metal Dichalcogenides and 2D Heterojunctions

Significant efforts have been made to tailor the thermal conductivity of transition metal dichalcogenides (TMDC) materials with promising thermoelectric performance. Starting with the MoS_2_, a continuously tuning of the thermal conductivity of suspended exfoliated (few layers) MoS_2_ flakes was demonstrated by exposure to a mild oxygen plasma [96]. The value of the in-plane thermal conductivity underwent a sharp drop down to values of the amorphous phase. In a recent experimental study, Li et al., showed that the in-plane thermal conductivity of monolayer crystals of MoS_2_ with isotopically enriched oxide precursors can be enhanced by ~50% compared with the MoS_2_ synthesized using mixed Mo isotopes from naturally occurring molybdenum oxide [97]. Furthermore, suspended polycrystalline MoS_2_ nanofilms with average grain sizes of a few nanometers also have been realized by using a new polymer- and residue-free wet transfer method, where a strong reduction of the in-plane thermal conductivity was found due to scattering of phonons on nanoscale grain boundaries [98]. The same group later systematically studied the impact of the grain orientation on the thermal conductivity of supported polycrystalline ultrathin films of MoS_2_. [99] The lowest *k* value (0.27 W m^−1^ K^−1^) was obtained in a polycrystalline sample formed by a combination of horizontally and vertically oriented grains in similar proportion. 

Different from MoS_2_, Chen et al. [22] studied the *k* anisotropy between the zigzag and armchair axes in suspended Td-WTe_2_ samples of different thicknesses. They found that as the 2D layer thickness decreases, the phonon-boundary scattering increases faster along the armchair direction, resulting in stronger anisotropy. Furthermore, recent studies showed that the thermal conductivity of monolayer WS_2_ (32 W m^−1^ K^−1^) [100] is comparable to the thermal conductivity of monolayer MoS_2_ and that is possible to achieve an ultra-low cross-plane thermal conductivity value (0.05 W m^−1^ K^−1^) in disordered WSe_2_ sheets [101]. Moreover, it was found that the thermal conductivity of a 45 nm thick TaSe_2_ film decreased almost 50% compared to its bulk value [102].

**Figure 6:**
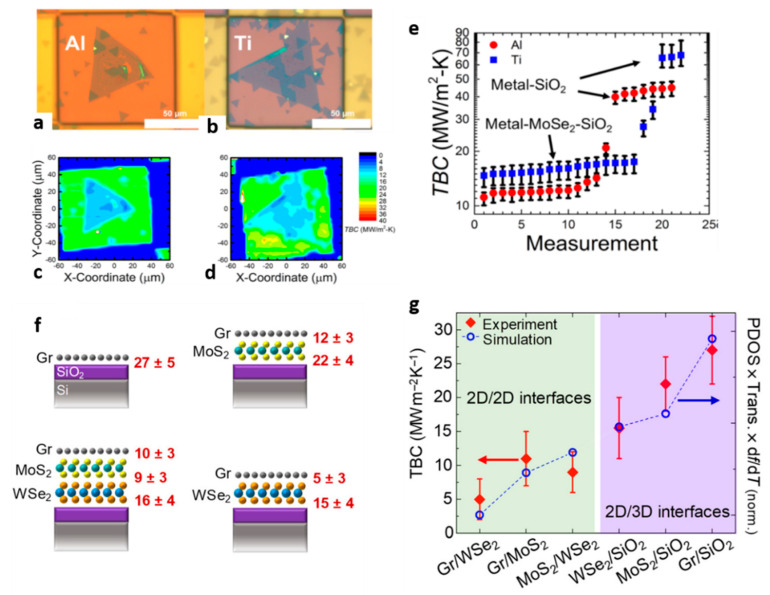
Phonon engineering in TMDC-based interfaces. Optical images showing the probed region of (**a**) Al–MoSe_2_–SiO_2_ and (**b**) Ti–MoSe_2_–SiO_2_ interfaces. (**c**,**d**) thermal boundary conductance (TBC) maps of the Al and Ti covered regions of the sample obtained by using time-domain thermoreflectance method (TDTR). (**e**) TBC values obtained at several positions across MoSe_2_ islands. (**f**) Schematics of TBCs measured across heterostructures consisting of graphene (Gr), Gr/ MoS_2_, Gr/WSe_2_, and Gr/MoS_2_/WSe_2_. (**g**) Measured TBC values of 2D/2D and 2D/3D (with SiO_2_) interfaces (red diamonds, left axis) and calculated values (open blue circles, right axis). The TBC were obtained by using single Laser Raman thermometry technique. (**a**–**e**) Reproduced with permission from [103]. Copyright American Chemical Society, 2019. (**f**,**g**) Reproduced with permission from [105]. Copyright American Institute of Physics, 2014.

Progress has also been made in engineering van der Waals (vdW) heterostructures or interfaces consisting of stacks of 2D monolayers with different materials in the in-plane and out-of-plane direction. Understanding and controlling the transport of thermal phonons in such nanostructures is necessary for the effective thermal management of devices based on TMDC materials. Therefore, there are currently significant experimental efforts towards the investigation of the interfacial thermal property of 2D heterojunctions. In particular, the majority of the experimental studies are focused on studying different ways to increase the TBC of 2D interfaces by forming heterojunctions consisting of TMDC materials and graphene or thin metal layers [103,104]. For instance, Brown et al. studied heat transport across different metal-TMDC heterojunctions [103]. They found a higher TBC value across Ti–MoSe_2_–SiO_2_ interfaces compared to Al–MoSe_2_–SiO_2_ due to the better interlayer adhesion between Ti and MoSe_2_ atoms. Figure 6a–d show the probed regions of these interfaces and thermal boundary conductance maps, respectively. A summary of the TBC values across different MoSe_2_-based interfaces are shown in Figure 6e. 

On the other hand, when thermal isolation is desired, the engineering of interfaces that exhibit high thermal resistance is highly desirable. For example, a recent study demonstrated that ultrathin trilayer heterostructure consisting of stacks of monolayer graphene, MoS_2_, and WSe_2_ exhibit ultra-high interface thermal resistance resulting in an effective thermal conductivity lower than air at 300 K [105]. A schematic of the different heterostructures investigated in this work and the measured TBC values are presented in Figure 6f,g, respectively.

## 3. Experimental Techniques for Thermal Characterization 

Numerous experimental techniques have been developed for micro- and nanoscale heat transport characterization, which can in general be categorized in to electrical and optical techniques. First, we review the most common electrical techniques, including the thermal bridge method, the electron-beam self-heating technique, the 3*ω*-method and scanning thermal microscopy (SThM). Then, we review optical techniques based on Raman spectroscopy, and on laser- thermo-reflectance, such as time-domain thermo-reflectance (TDTR), frequency domain thermo-reflectance (FDTR), and the thermal transient grating (TTG) method. We discuss the main limitations of these techniques, pointing out the main challenges for thermal investigations in low-dimensional structures. 

### 3.1. Electro-Thermal Techniques

#### 3.1.1. Suspended Thermal Bridge Method

**Figure 7:**
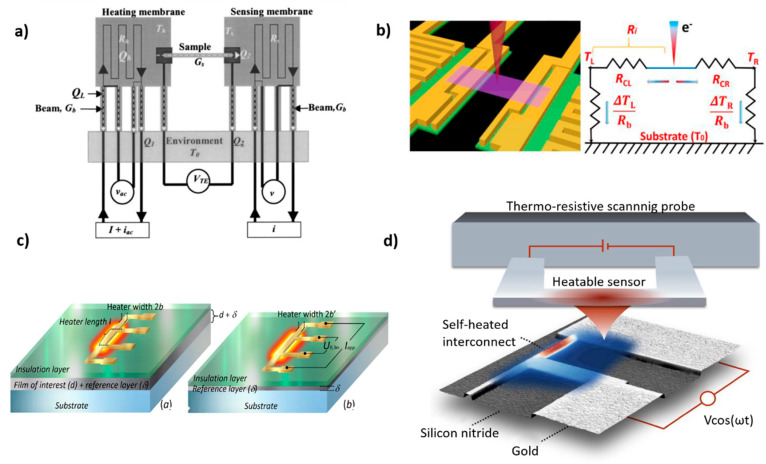
Electrical thermal characterization techniques. Schematic representations and thermal resistance circuits of (**a**) the electro-thermal bridge and (**b**) the electron beam self-heating technique. (**a**) Reproduced with permission from [107]. Copyright The American Society of Mechanical Engineers, 2003. (**b**) Reproduced with permission from [124]. Copyright Elsevier B.V., 2018. (**c**) Schematic of a three-omega heater deposited on a sample of interest with thickness d and small reference of thickness δ (left image) and a reference sample with thickness δ (right image). (**d**) Schematic illustration of the dual-sensing technique applied in a self-heated gold interconnect. Reproduced with permission from [159]. Copyright Nature Springer, 2016.

The thermal bridge technique is based on a microdevice consisting of two suspended silicon nitride (SiN_x_) membranes, which are patterned with metal thin lines (Pt resistors). The resistors are electrically connected to contact pads by four Pt leads and used as microheaters and thermometers, providing Joule heating and four-probe resistance measurements, respectively (see Figure 7a). The sample is placed between the two membranes and bonded to Pt electrodes while the heat transfer in the suspended sample is estimated by considering the generated Joule heating on the heated membrane and the temperature rise on the sensing membrane. This method offers high temperature resolution ~0.05 K [106,107] in a temperature range from 4 to 400 K due to the high accuracy of the Pt thermometers and direct temperature calibration. The experimentally measured thermal conductance *G* and thermal conductivity *k* are obtained from the equations *G* = 1⁄*R_tot_* and *k* = *L*⁄(*AR_tot_*), respectively, where *R_tot_* is the total measured thermal resistance, *L* is the length of the sample and *A* the cross section area of the sample. Here, *R_tot_* is the total thermal resistance of the full system, which includes the thermal resistance of the suspended sample, the thermal resistance contribution from the part of the sample that is connected with the membranes, the internal thermal resistances of the two membranes, and the additional thermal resistance contribution from part of the membranes which are connected with the heater/thermometers. This method was first introduced by Kim et al. to measure the in- plane thermal conductivity of suspended multi-walled nanotubes [106]. Since then, it has been used to measure the thermal conductivity of various materials, including nanofilms [108,109], 2D materials, such as graphene [77,110,111,112,113], boron nitride [3], and TMDC materials [96,114].

However, there are still some technical challenges that need to be addressed. The primary challenge is the accurate estimation of the thermal contact resistance components that inevitably contribute to the measured *R_tot_*. The first is the thermal contact resistance (*R*_*c*,*f*_) between the two ends of the suspended sample and the SiN_x_ membranes [108,109]. The estimation of this resistance requires the use of a fin resistance model, as reported elsewhere [113,115]. Another component of *R_tot_* is the thermal contact resistance between sample-membrane interface and thermometer (*R_c_*_,*m*_), which originates from the non-uniform temperature distribution on the heating membrane. *R_c_*_,*m*_ can be ignored, only when a uniform temperature distribution in the membrane can be assumed, i.e., when the thermal resistance of the suspended sample is large compared to the internal thermal resistance of the membrane. However, this is not the case for high thermal conductivity materials, such as graphene and carbon nanotubes. For instance, Jo et al. re-analyzed heat transport results reported in CVD single-layer graphene samples and found that such extrinsic thermal contact resistances contribute up to ~20% to the measured thermal resistance [113].

To overcome these difficulties, numerical heat transfer calculations have been conducted to estimate the exact temperature rise at the contact points between sample and heated membrane [3,77,116,117]. Moreover, several recent works reported the use of resistance line thermometers instead of a serpentine Pt thermometer in order to reduce the size of the temperature measurement region (between heater/sensor and contact point) [118,119]. Based on numerical heat conduction calculations, it has been found that this approach can reduce the contribution of *R*_*c*,*m*_ to about 30–40% compared to the *R*_*c*,*m*_ values that correspond to serpentine resistance thermometer devices [113]. Other approaches have been suggested to reduce *R*_*c*,*m*_ and improve the membrane temperature uniformity, such as adding high *k* materials to the membranes [120]. Furthermore, recent studies showed that the use of an integrated device fabricated from the same device layer as the membrane minimizes the thermal contact resistance between sample and membrane [36,121].

Other difficulties in this technique are related to the device fabrication and the sample transfer, which is technically challenging and time consuming. The transfer of exfoliated 2D materials to the thermal bridge structure is usually performed by a dry transfer method, which usually results in polymer residues, defects and rough edges on the sample surface that significantly affect the measured total thermal resistance [112,122]. The suspended thermal bridge method is applicable within the temperature range from 4 to 400 K. For sub-Kelvin measurements, a more sophisticated technique based on the tunnel current in a normal-metal-insulator-superconductor junction has been proposed [123], with the potential to operate down to 1 mK. 

#### 3.1.2. Electron Beam Self-Heating Technique

A new method for thermal characterization has been recently proposed, namely, electron-beam self-heating technique, which provides direct measurements of *R_c_* and *k* and overcomes the previously described limitations of the thermal bridge method [124]. Figure 7b shows a schematic of this technique, where a scanning electron beam is used as a heating source while the two suspended membranes act as temperature sensors. During the scanning of the focused electron beam along the length of the sample, a part of the electrons energy is absorbed at each position of the sample, creating local hot spots. The generated heat flux from the local spots flows towards the two membranes and rises their temperature while the thermal conductivity of the sample can be calculated by the equation *k* = *A/*(*dR*⁄*dx*), where *A* is the cross-sectional area of the sample, *R* is the measured thermal resistance from one membrane to the heating spot and *x* is the distance between membrane and heating spot. 

The main advantage of this technique is that the measured *R* contains the diffusive thermal resistance of the suspended part (*R_d_*) and the thermal contact resistance between the suspended sample and contact electrodes (*R_c_*), given by the equations: *R* = *R_d_* + *R_c_*, with *R_d_* = *L*/*ktW* and *RW* = *L*/*kt* + *R_c_W*, where *k*, *L*, *t*, and *W* are the thermal conductivity, length, thickness, and width of the suspended sample, respectively. *R_d_* decreases with increasing t and decreasing *L* and *R_c_* can be derived by taking the limit of *L*/*t*→ 0. However, in general, the spatial resolution is limited by the heating volume within the sample rather than the spot size, as it is the case in laser-based techniques. Therefore, the spatial resolution of this technique depends on the investigated materials properties [125]. The electron-beam self-heating technique has been used in recent works to measure the thermal conductivity and thermal resistance of suspended Si and SiGe nanowires, MoS_2_ ribbons [56,125,126], and the interfacial thermal resistance between few-layer MoS_2_ and Pt electrodes [96].

The primary difficulty in this technique is to generate sufficient temperature gradients from the electron beam spot to the two membranes, in particular in thin samples where the absorbed electron energy is relatively low. The low temperature rise at the sensors leads to a weak signal with low signal-to-noise ratio that is difficult to detect. Furthermore, this technique requires high-quality samples with flat and clean surface since the electron beam is strongly affected by defects, rough edges, and polymer residues that result in an increased error in the acquired thermal resistance signal. Optimization methods have been discussed related to the use of better electronics and data acquisition system with more sensitive, stable and high-precision signal processors and amplifiers [124]. Finally, Monte Carlo simulations have suggested that the enhancement of the acquisition signal can be achieved by modifying the acceleration voltage and spot size of the incident electron beam [116].

#### 3.1.3. Conventional Three-Omega Method

The conventional three-omega (3*ω*) method is based on the measurement of the third harmonic voltage of a thin metal line deposited on the material to be measured. The metal line serves both as the heater and the thermometer. This technique is an electro-thermal method widely used to determine the thermal conductivity of solids [127], liquids [128,129], and gases [130]. The experiment consists of applying an alternating current, *I_app_*(*t*) = *I*_0_cos(*ωt*) (where *I*_0_ is the current amplitude, *ω* is the angular frequency, i.e., *ω* = 2*πf* and *f* is the modulation frequency), to metal line (wire) deposited onto the sample surface. Due to the Joule heating, the temperature across the metallic strip (or 3*ω* -heater) oscillates with a frequency 2*ω* given by:

(1) $$\Delta T_{2\omega }=\frac{2U_{3\omega }}{\beta U_{0}}\approx \frac{2U_{3\omega ,\;rms}}{\beta U_{\omega ,\;rms}}$$

where *U*_0_ is the measured voltage of the wire, *U*_3*ω*_ is the three-omega voltage, i.e., the third harmonic component of the oscillating voltage and *β* is the temperature coefficient of the electrical resistance of the strip with *R*(*T*) = *R*_0_ (1 + *β*Δ*T*). Since the *U*_3_*_ω_* is at least three orders of magnitude smaller than the first harmonic (*U*_1*ω*_), a lock-in technique is required. The thermal fluctuation can therefore be obtained from the 3*ω* component in terms of root mean square quantities (rms). It is important to note that the noise of the whole 1*ω* signal is in the same order as the 3*ω* signal itself. Then, it is advisable to not measure *U*_3*ω*_ directly but rather with a passive circuit. Once the relationship between the Δ*T* and *U*_3*ω*_ is known, the thermal conductivity can be obtained by solving the transient heat equation for a finite width line heater, deposited onto a semi-infinite substrate. The temperature rise is given by:

(2) $$\Delta T_{2\omega }=\frac{P}{lk\pi }\int_{0}^{\infty }{\left(\frac{\sin\left(xb\right)}{xb}\right)}^{2}\frac{dx}{\sqrt{x^{2}+iq^{2}}}$$

where *P* is the applied power, *b* and *l* are the half-width and the length of the heater, respectively, *q* ≡ 1/*λ* = $\sqrt{2\omega /\alpha }$ is the inverse of the thermal penetration depth (*λ*), *α* is the thermal diffusivity, and *i* is the imaginary number. Equation (2) does not have an analytical solution, however, Cahill [127] showed that for *λ* >> *b* the heater can be approximated as line source. The upper limit of the integral can be replaced by 1/b and the sinusoidal term sin(*xb*)/(*xb*) → 1 in the limit of *b* → 0 and analytical solution is given by:

(3) $$\Delta T_{2\omega }\approx \frac{P}{2lk\pi }\left(-\ln\left(2\omega \right)+\ln\left(\frac{k}{\alpha b^{2}}\right)+2\gamma \right)-\frac{P}{4kl}i$$

where *γ* is constant. Finally, the *k* can be extracted from the slope of the real part of Δ*T*_2*ω*_ vs. ln(2*ω*):

(4) $$k\approx \frac{P}{2\pi l}{\left(\frac{d\left(\Delta T_{2\omega }\right)}{d\ln\left(2\omega \right)}\right)}^{-1}$$

This approximation is known as the slope method. For a film on a substrate, the estimation of *k* is carried out using the differential method [131,132]. To apply this method, the film has to have a *k* smaller than those of the substrate one and the heater width has to be larger than the film thickness. Under these conditions, it is possible to model the film as a frequency independent thermal resistance assuming that the heat flows cross-plane from heater to the substrate. The cross-plane thermal conductivity (*k*_⊥_) of a film on a substrate is given by:

(5) $$k\approx \frac{Pd}{2lb}\frac{1}{\Delta T_{f+s}-\Delta T_{s}}$$

where Δ*T*_*f*+*s*_ and Δ*T_s_* are the temperature rise of the film-substrate and substrate systems, respectively. From Equation (5) is evident that for each film-on-substrate measurement, it is necessary to create and measure at least two samples, i.e., one sample containing the film of interest and another with the substrate alone for calibration. To avoid any impact of the interface thermal resistance, it is advisable to deposit a small layer on the substrate to be used as reference (Figure 7c). The second sample is used to account for any impact of the interface thermal resistance in the measured temperature rise. 

This approach is mainly sensitive to *k*_⊥_. However, if the heater width is smaller than the sample thickness *d* (2*b* ≤ *d*), the heat flux will spread two-dimensionally with in- plane and cross-plane components. In this regime the stripe is sensitive to the in-plane (*k*_‖_) and cross-plane components of thermal conductivity and the temperature rise is given by [133]:

(6) $$\Delta T=\frac{-P}{\pi lk_{⊥,1}}\int_{0}^{\infty }\frac{1}{A_{1}B_{1}}{\left(\frac{\sin\left(bx\right)}{bx}\right)}^{2}dx,\;\mathrm{with}$$

(7) $$A_{j-1}=\left(A_{j}\frac{k_{⊥,\;j}}{k_{⊥,j-1}}\frac{B_{j}}{B_{j-1}}-\tan h\left(\varphi_{j-1}\right)\right)/\left(1-A_{j}\frac{k_{⊥,\;j}}{k_{⊥,\;j-1}}\frac{B_{j}}{B_{j-1}}\tan h\left(\varphi_{j-1}\right)\right),\;j=2,\;3,\;\ldots n$$

(8) $$B_{j}={\left(k_{\|,j}/k_{⊥,j}x^{2}+2i\omega /\alpha_{⊥,j}\right)}^{1/2}$$

(9) $$\varphi_{j}=B_{j}d_{j}$$

where the subscript *j* corresponds to the *j*th layer and *n* is the last layer (substrate). For the substrate (*j* = *n*) three approximations can be considered: (i) semi-infinite layer (*A_n_* = −1), finite thickness (*d_n_*), and (ii) adiabatic (*A_n_* = −tanh(*B_n_ d_n_*)), or (iii) isothermal (*A_n_* = −1/tanh(*B_n_ d_n_*)) boundary conditions.

Another approach to measure the in-plane thermal conductivity is the 3*ω*-Völklein method [134,135]. In this method, the 3*ω*-heater is patterned in the center of a suspended film or membrane. As the thermal sink is located at the edge of the structure, the in-plane thermal flux is ensured and, consequently, the temperature rise is governed by *k*_‖_. 

Additionally, Lu et al. showed that it is possible to extract the specific heat capacity and the thermal conductivity of filament- (rod-) like sample using the self-heating 3*ω*-method [136]. In this approach the sample is connected to four metals pads similar to a standard four-probe resistance measurement. The two outer connectors are used to pass an electrical current and the two inner pads measure the voltage. Three important modifications are added to this approach: (i) the sample in between the two voltage probes has to be suspended to allow temperature fluctuations; (ii) all the pads have to be highly thermal conductive to be used as heat sink of the sample to the substrate; and (iii) the measurement has to be carried out in vacuum and shielded at the same temperature than the substrate to minimize the radial heat loss through gas convection (or air conduction) and thermal radiation, respectively. In such configuration, the authors solved the one-dimensional heat equation of wire heated by an AC current and connected to an infinity heat sink from the voltage pads. For low frequency limit (<1 kHz), they found that the thermal conductivity can be described in terms of the 3*ω*-voltage as follows:

(10) $$U_{3\omega ,\;rms}≅\frac{4I_{0}^{3}R\beta }{\pi^{4}k}\frac{l}{S}$$

where *S* is the cross section of the sample, *I*_0_ is the current amplitude, *R* the electrical resistance, *β* is the temperature coefficient of the filament and *l* the length of the sample measured from the voltage (inner) pads, while for high frequency they found that the 3*ω*-voltage is sensitive to the volumetric specific heat (*C_V_*) as follows:

(11) $$U_{3\omega ,\;rms}≅\frac{I_{0}^{3}R\beta }{4\omega C_{V}lS}$$

Using this approach Lu et al. measured the thermal properties of platinum wires and multiwalled carbon nanotubes. Later several researchers used the same approach to measure the thermal properties of Si nanowires, multi- and single-walled carbon nanotubes [137,138], and nanoporous Si films [121] among others.

In general, the 3*ω*-heaters are patterned by photolithography using titanium, gold, platinum, or aluminum for the metallic layer. Depending on the electrical conductivity of the sample, an additional oxide layer deposition is required to ensure the electrical insulation of the heater. The deposited metallic strip is composed of four pads connected by pins to the narrow heating wire. The width of the heating line is defined as 2*b* and the length as *l*, the latter being determined by the distance between the inner pads. The outer two pads are used to apply the AC electrical current that generates the Joule heating (*I_app_*). The inner two pads are used to measure the voltage (*U*_0,3*ω*_), which contains the third harmonic component (see Figure 7c).

For bulk systems, the determination of *k* using the 3*ω*-method is straightforward. The main limitation comes from the fabrication of the 3*ω*-heaters and the growth of an insulation layer for electrically conductive substrates. For the case of thin films, the method is most sensitive if the *k* of the film is much smaller than the substrate. Borca-Tasciuc et al. [133] showed that the error in the estimation of the thermal conductivity of the film scales as (*k_film_*/*k_substrate_*)^2^. For films with thermal conductivities of the order of or larger than the substrate, the effect of the two-dimensional heat spread must be taken into account, i.e., the temperature rise has to be estimated using Equation (6). Other limitations of this technique include the impact of the surface roughness, i.e., a rough surface may lead to the breakage of the thin deposited wire deposited on to it.

#### 3.1.4. Scanning Thermal Microscopy

Scanning thermal microscopy (SThM) is an atomic force microscopy (AFM)-based technique that has been extensively used for quantitative nanothermal measurements, including temperature [139,140,141,142,143] and thermal conductance [144,145,146,147,148] measurements. Depending on the material under investigation and the required material property that needs to be measured different tips and modes of operations have been implemented. For thermal measurements, a typical SThM setup consists of a sharp tip acting as a heater/temperature sensor, a cantilever with a feedback system (e.g., an electromechanical system) to control the tip-sample interaction and several electronic components. 

For temperature measurements the SThM setup is used in a passive mode of operation, where the tip acts as a thermometer while an external heat source, e.g., electrical contacts or laser, provides Joule heating to the investigated structure. Passive SThM requires low power bias applied to the tip sensing element to avoid self-heating. In the case of resistive thermometers, the temperature measurements rely on the temperature dependence of the electrical resistance of the tip, which is given by *R_p_* (*T*) = *R*_0_ (1 + β(*T* − *T*_0_)), where *R*_0_ is the electrical resistance of the probe at a reference temperature *T*_0_ and β is the temperature coefficient of the electrical resistance. In the case of metallic contacts, the local temperature at the sample surface can be obtained also by measuring the thermoelectric voltage at the point contact [140]. Nevertheless, the main challenge in temperature measurements is to accurately relate the sensor signal to the temperature of the surface. This is a difficult task due to the fact that non-equilibrium processes take place at nanoscale contacts and the temperature distribution across the tip-sample interface appears discontinuous. In particular, the heat flux-related signal acquired from the temperature difference between tip-sample, is also influenced by an unknown thermal contact resistance [149], which increases as the tip-sample contact size decreases. In addition, topography related artifacts due to modulation of the effective tip-sample contact area result in additional errors in the measured temperature. Consequently, temperature measurements with nanoscale resolution are not straightforward. 

To overcome these issues different methods have been developed, such as the null-point method [150] and the dual-sensing technique [139]. The former is based on creating a thermal equilibrium between tip and sample surface to eliminate the heat flux signals at the thermal contact point and minimize the influence of tip-sample contact resistance in the temperature measurements. Similarly, in the dual-sensing technique the authors demonstrated a way to separate variations of tip-sample thermal contact resistance from sample temperature variations, eliminating in parallel topography related artifacts. Figure 7d show an illustration of this technique applied to a metal interconnect, where a sinusoidal voltage ~*V*cos(*ω*t) was used to modulate the sample temperature. Then, the sample temperature field was extracted by simultaneously probing a time-dependent and a time-averaged heat flux signal between the hot tip (red colored) and the sample. 

For thermal conductance measurements, the resistive element of the probe is used additionally as a heater to induce local heating at the tip-sample junction. The measured heat flux signal depends on both the tip-sample temperature difference and tip-sample thermal resistance (*R_ts_*) and is equal to *Q* = (*T_t_* − *T_s_*)/*R_ts_*, where *T_t_* is the temperature of the tip, usually controlled by applying a current or voltage to the tip, and *T_s_* is the temperature of the sample. Then, the *R_ts_* can be extracted from the thermal resistance change upon tip-sample contact, as $R_{ts}={\left(R_{th\left(c\right)}^{-1}-R_{th\left(out\right)}^{-1}\right)}^{-1}$. The measured *R_ts_* depends on the sample thermal conductivity and tip-sample interfacial thermal resistance and is usually described by a series of resistors, as *R_ts_* = *R_t_* + *R_c_* + *R_spr_*, where *R_t_* is the thermal resistance of the tip, *R_c_* is the thermal contact resistance between tip and sample and *R_spr_* is the thermal spreading resistance in the sample. The contributions of such resistive components on the measured thermal resistance are usually determined taking into account the calibration of the tip and analytical or numerical models of the heat spreading according to the geometry of the tip-sample system. More details regarding the quantification of these components can be found elsewhere [151,152]. 

The main difficulty in thermal conductance measurements using the SThM technique is to minimize variations of the effective tip-sample contact area in order to avoid topography-related resistance modulations. Thus, a careful comparison between topography and thermal resistance data is required. This difficulty also complicates the direct comparison of thermal transport data between different SThM setups and thermal probes. In addition, when thermal measurements performed in ambient conditions, parasitic heat effects resulting from the heat transfer through the liquid meniscus and air must be taken into account [134]. Measurements in high vacuum conditions, accurate estimations of the tip-sample contact area, and modelling of the tip-sample system have helped to overcome the above difficulties [151].

Therefore, in contrast to the previously described techniques, SThM does not provide direct access to the thermal conductivity of the investigated sample. The determination of the thermal conductivity requires additional modelling, strong assumptions, and several calibration steps on reference samples [153,154]. Despite these difficulties, recent studies have successfully used the SThM technique to quantitatively determine the thermal conductivity of 2D materials, such as graphene [143,155,156]. However, SThM is considered to be ideal to investigate heat transport at nanoscale contacts and interfaces with sub-nW and sub-10 nm heat flux and thermal spatial resolution, respectively. The SThM technique has been employed recently to investigate heat transfer in semiconductor nanostructures, e.g., nanowires [115,157,158,159], supported thin films [145,160,161] and 2D materials [142,143,144,146,148,154,155,156,162,163]. For instance, recently El Sachat et al. [144] performed high-vacuum SThM measurements to experimentally probe the transition from ballistic to diffusive thermal transport in suspended single-layer graphene. The authors also revealed that graphene’s surface quality, e.g., defect concentration and surface contamination, as well as morphology, have crucial influence on in-plane thermal conductance measurements, and need to be included to extract the intrinsic transport properties of graphene. SThM also has been successfully employed to reveal hot spots in graphene electronic devices and self-heating 2D heterojunctions by directly mapping the spatial distribution of the generated steady-state temperature rise [142,143,163]. Furthermore, novel SThM configurations have been developed the last years to simultaneously study thermal and thermoelectric transport on a nanometer scale [164,165,166], revealing important effects such as local Joule heating, Seebeck and Peltier effects in graphene and nanowire heterostructures. Such measurements gave further insight into phonon transport at the nanoscale and showed the great advantage of using thermal characterization tools with thermal and topographic mapping capabilities.

### 3.2. Optical Techniques

#### 3.2.1. Opto-Thermal Raman Spectroscopy and Thermometry

Raman spectroscopy is an optical technique dedicated to the study of molecular vibrational modes and phonons in solids. The technique analyzes the inelastically scattered light of a monochromatic laser beam that interacts with a material. The oscillating electromagnetic field of the incident light induces an oscillating electric dipole moment, which acts as a radiation source causing the Raman scattering. Each material or solid crystal has its own set of characteristic molecular vibrations and phonons that depend on the nature of the chemical bonds and the crystal structure. This technique is commonly used as a tool for elementary and structural characterization of the materials. In addition, small changes in the crystal structure induced by: embedded strain, thermal expansion, sample compositional and structural disorder, impurities and contamination of the sample, as well as the presence of pseudo-phases and deformation of the material can be also detected using this technique [167,168,169,170].

Another particular application of Raman spectroscopy is the determination of the local temperature of the material under analysis and, consequently, its thermal properties. In a crystal structure, an increase in temperature displaces atoms from their equilibrium positions which, in turn, results in an overall volumetric expansion of the lattice. The expansion of the lattice induces a change in the interatomic forces and, as a result, the Raman modes shift to lower wave numbers as the temperature increases. Similarly, the linewidth of the Raman spectrum is broadened as the temperature increases as consequence of the temperature-dependence of the phonon lifetime. Moreover, the Stokes to anti-Stoke ratio is also modified due to the temperature dependence of the phonon population. Thus, once the temperature dependence of the Raman spectra is known, any of these parameters can be used as a local thermometer [171]. For example, if the redshift of the Raman mode is used as a thermometer, the local temperature of a focused spot can be easily obtained by fitting the spectral position of the mode, given the previous calibration of its spectral position with temperature, which, in general, exhibits a linear dependence. 

**Figure 8:**
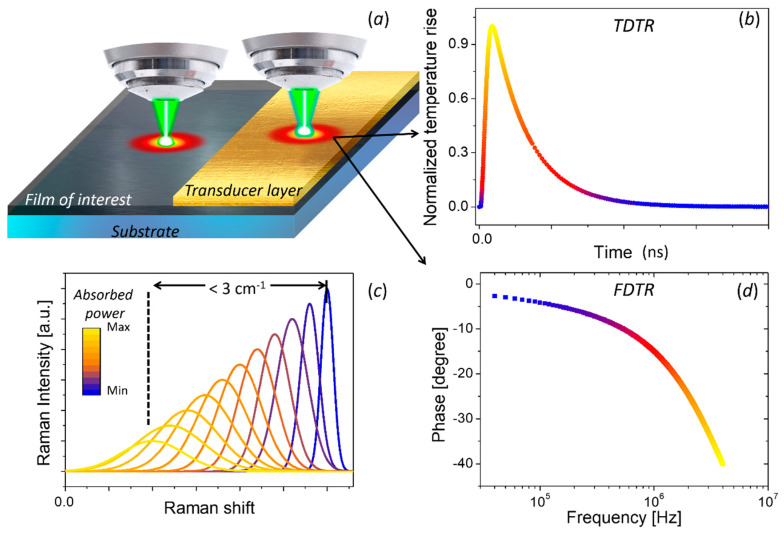
Optical thermal characterization techniques. (**a**) Schematic representation of Raman thermometry (left) and thermoreflectance (right) technique. Typical recorded signal using: (**b**) Time domain thermoreflectance (TDTR), (**c**) Raman thermometry, and (**d**) frequency domain thermoreflectance (FDTR).

When a given material absorbs wavelength of laser light, the incident power will induce local heating and, consequently, a red-shift of the observed Raman signal (see Figure 8a,c). The temperature rise in the illuminated region will depend on the thermal properties of the material. Alternatively, if a material is heated by an external source, e.g., by passing an electrical current or illuminating with a second laser, the temperature gradients produced by this source can be also measured using the redshift of its Raman signal. 

Once the thermal map or the local temperature rise is measured, the thermal properties of the sample can be extracted with a suitable heat diffusion model. For bulk materials the three-dimensional heat equation has to be solved considering a Gaussian power source [172,173]. For thin films on a substrate the problem is analytically more complicated [174,175,176]. While for systems with large optical absorption an analytical expression of the thermal conductivity is given by [177]:

(12) $$k=\left(\frac{\partial \omega }{\partial T}\right)\left(\frac{1}{4\sigma \sqrt{\pi }}\right){\left(\frac{\partial \omega }{\partial P_{a}}\right)}^{-1}$$

where ∂*ω*⁄∂*T* is the slope of the peak position (ω) vs. temperature, σ the spot size, and ∂*ω*⁄∂*P_a_* is the slope of the peak position vs. the absorbed power. 

The main limitation of this technique is the requirement that the material have Raman active modes, which is not the case for metals or centrosymmetric materials. For materials with weak active Raman modes this method can be very time consuming, especially for measurements at low power. Other important limitations of this technique is the weak temperature dependence of the Raman modes. In general, a linear temperature-dependence of the peak position is observed as *ω*(*T*) ≈ *ω*_0_ + *χ_T_*Δ*T*, with a slope *χ_T_* of the order of ~ −10^−2^ cm^−1^ K^−1^. Considering that a state-of-the-art Raman spectrometer has a frequency resolution ~0.5 cm^−1^ and the peak fitting can enhance it to ~0.25 cm^−1^ [129], a detectable temperature rise has to be Δ*T* ≥ 20 K. This high temperature rise has a direct impact in materials with large temperature dependence of its thermal conductivity, *k*(*T*). For example, the temperature-dependence of the thermal conductivity of bulk silicon varies as [178]:

(13) $$k\left(T\right)=150\left[WK^{-1}m^{-1}\right]{\left(\frac{T}{300\left[K\right]}\right)}^{-1.65}$$

On the other hand, the slope of the LO mode frequency against temperature in Si varies as *χ_T_* ~ 2 × 10^−2^ cm^−1^ K^−1^ [20]. Then, a Δ*T* = 20 K above room temperature, i.e., *T* = 320 K, will shift the peak position by only 0.4 cm^−1^, i.e., just above the detection limit, but it will reduce *k* by 10% due to its temperature-dependence. Another important limitation of this technique is the need of measurement of the absolute absorbed power. The laser absorptivity for supported films or any nanostructure is very difficult to be determined and it could induce a large error on the thermal conductivity determination. 

The first studies of the thermal properties of single-layer graphene were conducted by Balandin et al. [179,180] using Raman thermometry. Since then, Raman thermometry has been used in a wide range of 2D materials [22,73,98,181,182,183,184,185], carbon nanotubes [186], nanowires [187,188,189], nanomembranes [190,191,192,193], and phononic crystals [24,33], among others. In general, the 2D material is transferred over a substrate, which was previously patterned with micro-holes and covered with metal layer to ensure a good thermal contact. The 2D material is suspended and the Raman laser is positioned at the center of the hole. Then, a Raman spectrum as a function of incident power is measured. The temperature rise is obtained from the previously calibrated Raman frequency shift and the thermal conductivity is obtained from numerical analysis. A deep and extended description of Raman-based technique for measuring thermal properties in graphene and related materials can be found in a recent review article by Malekpour and Balandin [194].

#### 3.2.2. Thermoreflectance-Based Techniques

The thermoreflectance methods are based on measuring changes in reflectivity (Δ*R*) induced by a change in the local temperature of a tested sample (Δ*T*) [195]. The basic concept consists of modulating the surface temperature of a sample by a pulsed laser (pump) and recording the changes of the temperature by monitoring the resulting changes in reflectivity with a second laser (probe). In general, the samples are covered by a metal layer (see Figure 8a) which acts as transducer with a well-known temperature dependence of its reflectivity for a given wavelength. In metals the temperature dependence of the reflectivity for a given wavelength can be explained in terms of: (i) free-electron-like behavior for infrared excitation (>1 μm), (ii) interband transitions for visible light (<1 μm), and (iii) collective oscillations, possibly in both regions [196]. 

Depending on the configuration, the change of reflectivity can be measured with respect to time (time-domain thermoreflectance, TDTR) or with respect to the modulated frequency (frequency-domain thermoereflectance, FDTR). TDTR measures the response of reflectance as a function of time delay between the periodic heat flux and the surface temperature (see Figure 8b) [197,198]. FDTR measures the phase lag between a periodic heat flux and the surface temperature over a range of heating frequencies (see Figure 8d) [199].

**Figure 9:**
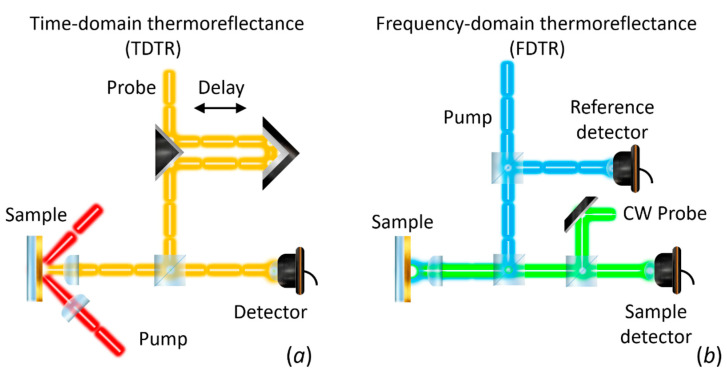
Schematic representation of thermoreflectance-based methods. (**a**) Time-domain and (**b**) frequency-domain thermoreflectance techniques.

The first report on the use of the thermoreflectance technique to measure thermal diffusivity dates back to 1986. Paddock and Eesley [197] described thermal diffusivity measurements of metals using picosecond transient thermoreflectance. In this method, two pulsed lasers are focused on a metal surface as shown in Figure 9a. A high-power (pump) laser induces an ultrafast heating of the surface, thus modulating its reflectivity. A low-power (probe) laser is focused on the heated spot and the reflected light is recorded by a photodetector. The measured signal is sent to a lock-in amplifier referenced to the frequency of the pump. The voltage output from the lock-in will be proportional to Δ*R*. By changing the delay line, it is possible to obtain Δ*R* as a function of optical probe-pulse time delay for a fixed frequency modulation (see Figure 8b).

Similarly, FDTR measures the reflectance response of the transducer layer as function of excitation frequency. The pump heats the surface sample periodically at a frequency f, and the probe beam is used to measure the change in reflectivity. A lock-in amplifier records the amplitude and the phase delay response of the reflected beam using the pump light as reference. The phase delay between the pump heating and the change in reflectivity, as measured by the probe beam, is typically used to determine the thermal diffusivity (see Figure 8d) since the amplitude at each frequency is affected by the frequency response of the detector and the cables [199]. Figure 9b shows a schematic representation of the FDTR using a CW laser as probe signal. Other configurations, including the use of a two pulsed lasers has been also reported [199].

In both TDTR and FDTR, the estimation of the thermal properties usually relies on a multilayer model developed by Cahill [154]. He solved a three-dimensional heat equation taking into account that the response of a new pulse should account for the previous pulse with a non-negligible value (“pulse accumulation” effect). Later, Schmidt et al. [199] extended the model including the impact of the thermal anisotropy and adapted it to FDTR. Using the latter model the thermal properties are determined from the best fit of the theoretical model to the experimental data by using the thermal unknown (e.g., thermal conductivity) as a free parameter. An extended description and discussion of the model can be found in ref. (Cahill 2004 [154], Schmidt 2009 [199] and Jiang 2018 [198]) Numerical code can be found in the webpage of Cahill’s group (https://cahill.matse.illinois.edu/software-and-data/).

The main limitation of the thermoreflectance technique is the need for very smooth surfaces. Otherwise the diffuse scattering of the reflected light precludes the measurement of the thermoreflectance signal. Additionally, an interfacial thermal resistance between sample-transducer, and in the case of layered structures between different layers, can impact the measured thermal properties of the investigated material. Schmidt et al. [122] and Cahill et al. [154] have developed an analytical heat transfer model where such interfacial thermal resistances can be estimated and included in the data analysis. In particular, the interface thermal conductance is treated by taking the limit as the heat capacity of a layer approaches zero and is defined as *G* = *k*_⊥_/*d*, where *k*_⊥_ is the cross-plane thermal conductivity and *d* is the layer thickness.

For graphene, it is well known that the *k* of a free-standing single-layer shows very high values in the range of 600–5000 W m^−1^ K^−1^ [76,179,200,201,202], but the supported and encased graphene exhibits a large reduction of *k* in the range of 50–1200 W m^−1^ K^−1^ [201] and <160 W m^−1^ K^−1^ [88], respectively. For supported and encased 2D-materials, the heat transfer is inhibited by phonon interactions at the interfaces. Another important limitation is its applicability to the analysis of in-plane properties of 2D materials, as the method cannot be effectively applied to measure in-plane thermal conduction of films with thicknesses below 20 nm [198].

#### 3.2.3. Thermal Transient Grating (TTG) Method 

**Figure 10:**
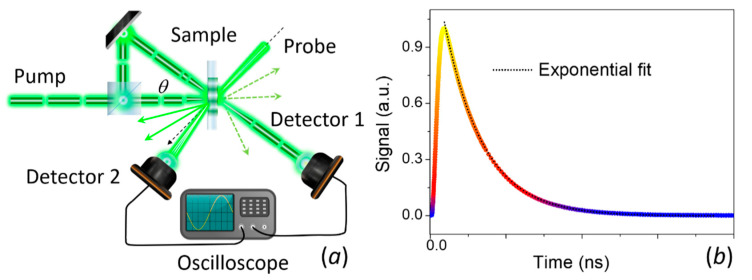
The thermal transient grating technique (TTG). (**a**) Schematic representation of TTG and (**b**) artistic representation of a typical signal.

The thermal-transient gradient method is also an optical technique primarily for measuring the thermal diffusivity [203,204] and acoustic properties [205,206] of materials. In this method an optical interference pattern is created by crossing two laser pulses of wavelength λ at an angle *θ*. The subsequent optical absorption will cause a spatially sinusoidal thermal grating with a period *L* = *λ*/ (2 sin(*θ*/2)). As a consequence, an optical phase and amplitude grating will be induced through the temperature dependence of optical properties of the material. A second laser (probe) is used to monitor the magnitude of this grating. If the probe diameter extends over many grating periods, the beam is diffracted by this pattern and the thermal diffusivity can be determined from the rate of the signal decay. A schematic representation of the setup is shown in Figure 10a. As the heat diffuses from the peak to the valley of the grating, the diffraction efficiency of the optical grating decreases and the signal intensity decays exponentially with time as *T*(*t*)~exp(−*q*^2^α*t*), where *q* = 2*π*/*L* is the grating wave vector and α is the thermal diffusivity (see Figure 10b).

One of the main advantage of the TTG method is the absence of metal layer acting as transducer. This not only simplifies sample preparation, but also reduces complexity in the analysis of thermal properties due to the absence of thermal contact resistances from the transducer layer. In addition, the thermal length scale can also easily be varied by changing the grating period, which is useful to ensure diffusive transport and/or observe non-diffusive phonon transport [207]. Finally, as the thermal grating is defined in the plane of the sample, in-plane thermal transport is always assured. The main drawback of the technique is the complexity of the setup itself that requires a well-trained operator. In addition, the signal of the probe beam is very weak due low efficiency of the diffraction. This limitation can be overcome by the heterodyne detection [205]. Moreover, the use of samples with flat surfaces becomes mandatory to avoid large diffuse scattering of the light. The typical experimental uncertainty calculated from the standard deviation from several measurements is 10–15% [208].

## 4. Summary and Perspectives

We have presented an overview of recent strategies for engineering the heat transport by phonons that have been applied to possible technologically-relevant materials, such as semiconductor nanostructures, like nanowires, superlattices, phononic crystals, and 2D materials with extraordinary electronic, optical, mechanical, and thermal properties. We reviewed and compared thermal characterization tools used to determine thermal properties of low-dimensional structures, pointing out their main advantages and limitations (see Table 1). Progress in material growth and fabrication has enabled the emergence of a vibrant research area of heat transport at the nanoscale, which presents a myriad of exciting phenomena such as access to thermal transport regimes beyond diffusive transport, i.e., ballistic and hydrodynamic [209,210], and to fundamental aspects of the heat transport that open new technical prospects such as ballistic cooling [211]. 

Although significant progress has been accomplished in thermal transport engineering and thermal characterization great challenges still remain. Heat dissipation in the nanoscale is still poorly understood owing to the described technical limitations of current characterization techniques and the high sensitivity of phonon states to the technological process involved in the fabrication of samples and devices, as well as during their integration in circuits. The majority of today’s electronic components, e.g., nanoscale transistors, consist of materials with multiple interfaces, nanoscale contacts and boundaries, thus key questions have to be addressed related to the interfacial thermal energy transfer and the heat transport at nanometer-sized contacts. In addition to the emerging need to understand heat dissipation in materials and devices, progress in nanoscale thermal characterization is necessary to investigate non-equilibrium thermal processes highly-localized in space. In these processes the temperature depends on the time scale of the measurement and the sensitivity of the sensor (thermometer), i.e., the study of dynamic effects in systems out of thermal equilibrium requires high temporal resolution (~*ps*). Although certain optical experimental techniques, such as ultra-fast laser-based thermo-reflectance techniques, fulfil this requirement, the in-plane thermal spatial resolution is limited by the diffraction limit (sub-μm) while the necessary use of metallic coating (transducer) brings up new issues about the phonon transmission across interfaces. Raman thermometry addresses the later since it can directly measure the temperature difference across an interface, however, the temperature resolution is material-dependent and limited by the spectrometer resolution. On the other hand, SThM provides high temperature and spatial resolution, which makes it ideal to study heat energy transfer between sub-micrometer layer interfaces. However, its low temporal resolution complicates the investigation of non-equilibrium effects. The capability to study thermal dynamic effects with submicrometer thermal spatial resolution will pave the way for the better understanding of the basic principles governing heat propagation, scattering processes on nanoscopic length scales and thermal transport across atomic-layer interfaces.

## Figures and Tables

**Table 1:** Summary of high-resolution thermal characterization methods.

			Resolution				
Method	Material Geometry	Measurement	Temperature	Spatial	Temporal	Imaging	Limitations
Suspended thermal bridge	Suspended 2D materials, thin films, NWs, etc	*k*_‖_	~50 mK	Mean value	-	No	Difficult sample preparation, influence of extrinsic thermal contact resistances
Electron beam self-heating	Suspended 2D materials, thin films, NWs, etc	*k*_‖_, *Rc*, TBC	~0.4 mK	~20 nm (heating volume dependent)	-	No	Limited to thick samples, difficult sample preparation
3w-method	Supported and suspended films	*k*_‖_, *k*_⊥_	Mean value	Mean value	-	No	For electrical conductive films, electrical insulation is needed
SThM	Supported and suspended 2D materials, films, NWs, bulk etc.	*R_ts_*, *T*	<5 mK	<10 nm	10–100 µs	Yes	No direct access to k, hard modelling is needed
Raman spectroscopy	Supported and suspended 2D materials, films, NWs, bulk, etc	*k*_‖_, *k*_⊥_, TBC	~2 K	~λ/2 nm	-	Yes	Assumptions to determine k, complex sample preparation for 2D materials
Two-laser Raman Themometry	Suspend membrane-based structures, 2D materials	*k*_‖_	~2 K	~λ/2 nm	-	Yes	Limited to suspended structures
Frequency domain thermoreflectance	Supported 2D materials and films	*k*_‖_, *k*_⊥_, TBC	Sub-100 mK	~λ/2 nm	Sub-ps	Yes	Deposition of a thin metal film (transducer) is required
Time domain thermoreflectance	Supported 2D materials and films	*k*_‖_, *k*_⊥_, TBC	Sub-100 mK	~λ/2 nm	<1 ns	Yes	Deposition of a thin metal film (transducer) is required
Thermal transient grating	Supported and suspended fims	α_‖_	Sub-100 mK	~50 μm	10’s ps	No	Limited to the efficiency of the diffraction pattern
